# Emotion dysregulation and suicidality in eating disorders

**DOI:** 10.1002/eat.23410

**Published:** 2020-11-18

**Authors:** Marianna Rania, Elin Monell, Arvid Sjölander, Cynthia M. Bulik

**Affiliations:** ^1^ Department of Health Sciences University Magna Graecia of Catanzaro Catanzaro Italy; ^2^ Center for Clinical Research and Treatment of Eating Disorders Mater Domini University Hospital Catanzaro Italy; ^3^ Department of Medical Epidemiology and Biostatistics Karolinska Institutet Stockholm Sweden; ^4^ Centre for Psychiatry Research, Department of Clinical Neuroscience Karolinska Institutet Stockholm Sweden; ^5^ Stockholm Health Care Services Region Stockholm Stockholm Sweden; ^6^ Department of Psychiatry University of North Carolina at Chapel Hill Chapel Hill North Carolina USA; ^7^ Department of Nutrition University of North Carolina at Chapel Hill Chapel Hill North Carolina USA

**Keywords:** depression, DERS, eating disorders, emotion dysregulation, suicidal ideation, suicide attempts, suicide risk

## Abstract

**Objective:**

Suicidality in eating disorders (EDs) is high, and identification of therapeutically targetable traits associated with past, current, and future suicidality is of considerable clinical importance. We examined overall and ED subtype‐specific associations among suicidal ideation, suicide attempts, and general and specific aspects of emotion dysregulation in a large sample of individuals with ED, at presentation for treatment and 1‐year follow‐up.

**Method:**

Using registry data from 2,406 patients, scores on the Difficulties in Emotion Dysregulation Scale (DERS) at initial registration were examined as predictors of recent suicidal ideation and self‐report lifetime suicide attempts. Associations were examined in the full sample and in each ED subtype. In 406 patients, initial DERS scores were examined as predictors of suicidality at 1‐year follow‐up.

**Results:**

Overall DERS was associated with suicidal ideation and suicide attempts, even when adjusting for ED psychopathology and current depression. Perceived lack of emotion regulation strategies showed unique associations with suicidal ideation and suicide attempts, both in the full sample and in most ED subtypes. Initial DERS was also associated with follow‐up suicidal ideation and suicide attempts, although this association did not remain when adjusting for past suicidality.

**Discussion:**

Results suggest that emotion dysregulation may be a potential mechanism contributing to suicidality in EDs, beyond the effects of ED psychopathology and current depression. Although the prevalence of suicidality differs across ED subtypes, emotion dysregulation may represent a risk trait for future suicidality that applies transdiagnostically. Results support addressing emotion dysregulation in treatment in order to reduce suicidality.

## 1 INTRODUCTION

Eating disorders (EDs) have amongst the highest standardized mortality ratios for death by suicide of any mental disorder (Chesney, Goodwin, & Fazel, 2014; Huas et al., 2013; Preti, Rocchi, Sisti, Camboni, & Miotto, 2011). In order to detect individuals at risk for suicide, address vulnerability factors in treatment, and prevent suicide, identification of traits associated with suicidal ideation and suicide attempts, hereafter referred to as suicidality, in this population is of considerable clinical importance.

Emotion dysregulation, characterized by difficulties in emotional awareness, clarity and acceptance, as well as difficulties managing emotions and refraining from impulsive behaviors when in distress (Gratz & Roemer, 2004), has been associated with suicidal behaviors in general and specifically in individuals with EDs (Gomez‐Exposito et al., 2016; Pisetsky, Haynos, Lavender, Crow, & Peterson, 2017). We explored associations between aspects of emotion dysregulation and suicidality in a large sample of patients across a range of ED subtypes.

Suicidality, including suicidal ideation, suicide attempts, and death by suicide, is common in EDs. Lifetime prevalence estimates for suicide attempts in EDs range broadly from 3.0% to 40% (Bulik et al., 2008; Crow et al., 2009; Franko & Keel, 2006; Pisetsky, Thornton, Lichtenstein, Pedersen, & Bulik, 2013; Runfola, Thornton, Pisetsky, Bulik, & Birgegård, 2014; Smith, Zuromski, & Dodd, 2018; Udo, Bitley, & Grilo, 2019), and both ED diagnosis (OR = 2.19) and ED symptoms (OR = 1.81) are associated with increased odds for later suicide attempts (Smith, Velkoff, Ribeiro, & Franklin, 2019). Data from an epidemiologic survey of US adults with lifetime DSM‐5 ED diagnoses (American Psychiatric Association, 2013) indicated even higher prevalence of lifetime suicide attempts, especially in individuals with anorexia nervosa binge‐eating/purging type (AN‐BP; 44.1%), followed by 31.4% for bulimia nervosa (BN), 22.9% for binge‐eating disorder (BED), and 15.7% for AN restricting type (AN‐R) (Udo et al., 2019). Suicidal ideation (i.e., thinking about, considering, or planning suicide) often precedes suicide attempt (Nock et al., 2008), but has received less research attention in EDs. Lifetime suicidal ideation occurs in about one third of individuals with EDs (Favaro & Santonastaso, 1997; Milos, Spindler, Hepp, & Schnyder, 2004; Swanson, Crow, Le Grange, Swendsen, & Merikangas, 2011), and a nationwide Swedish ED treatment registry study indicated the highest prevalence estimates in BN and BED (36–39%) and the lowest in AN and other specified feeding and ED (OSFED; 26–30%). The presence of purging behavior is associated with greater risk of suicidality (Andersén & Birgegård, 2017; Swanson et al., 2011).

The mechanisms influencing the co‐occurrence of EDs and suicidality are unclear. In fact, in the national US epidemiologic survey, between 17–45% reported a suicide attempt before the onset of their EDs (Udo et al., 2019). Possible explanations for this co‐occurrence include comorbid psychiatric disorders such as depression, bipolar disorder, and substance abuse (Cliffe et al., 2020; Franko & Keel, 2006; Smith, Zuromski, et al., 2018), psychological and personality traits (Bulik et al., 2008; Milos et al., 2004; Pisetsky et al., 2015), self‐image (Andersén & Birgegård, 2017), early cognitive schema (Portzky, van Heeringen, & Vervaet, 2014), interoceptive deficits (Smith, Forrest, & Velkoff, 2018), alexithymia (Carano et al., 2012), and impulsivity (Sagiv & Gvion, 2020). Some of these traits are subsumed under the multidimensional concept of emotion dysregulation (Gratz & Roemer, 2004). Although emotion dysregulation has received substantial research attention in relation to both EDs (e.g., Monell, Clinton, & Birgegård, 2018; Prefit, Candea, & Szentagotai‐Tatar, 2019) and suicidality (Bradley et al., 2011; Harris, Chelminski, Dalrymple, Morgan, & Zimmerman, 2018; Pisani et al., 2013; Rajappa, Gallagher, & Miranda, 2012; Weinberg & Klonsky, 2009), its role in their co‐occurrence is less clear.

Using the Difficulties in Emotion Regulation Scale (DERS) (Gratz & Roemer, 2004) in 122 patients with bulimic symptoms (AN‐BP, BN, BED, OSFED), Gómez‐Expósito and colleagues reported that individuals with previous suicide attempts exhibited higher impairment than patients without suicide attempts in most aspects of emotion dysregulation, except difficulties in emotional awareness (Gomez‐Exposito et al., 2016). Similarly, Smith and colleagues examined emotion dysregulation in 100 patients with a range of EDs (AN, BN, BED, OSFED, unspecified feeding and ED [UFED]) and found higher perceived lack of adaptive emotion regulation strategies in those who reported previous suicide attempts than in those who did not (Smith, Forrest, et al., 2018). Conversely, Pisetsky and colleagues reported no significant differences in aspects of emotion dysregulation between ED patients with and without suicide attempts (*N* = 110 patients; AN, BN, BED, OSFED), although a trend with small effect size emerged for higher levels of difficulties with impulse control and emotional clarity in those with lifetime suicide attempts (Pisetsky, Haynos, et al., 2017). Small sample sizes precluded subgroup analyses and these studies were cross‐sectional.

The present study extends the existing literature by exploring associations between suicidality and emotion dysregulation in a large, well‐characterized sample of individuals with a range of EDs. Specifically, we examined associations between general and specific aspects of emotion dysregulation assessed at initial registration for treatment and recent suicidal ideation and lifetime suicide attempts. As exploratory analyses, we also investigated ED subtype‐specific associations between emotion dysregulation at initial registration and suicidality. Lastly, we explored whether emotion dysregulation at initial registration predicted suicidality at 1‐year follow‐up in a subset of the sample with available data.

## 2 METHOD

### 2.1 The stepwise database

The sample was drawn from the Stepwise database that includes data from patients entering ED specialist treatment at any of 45 treatment units in Sweden since 2005 (Birgegård, Björck, & Clinton, 2010). Stepwise inclusion criteria include referral to an ED treatment unit, a DSM‐IV ED diagnosis (American Psychiatric Association, 2000), and established intent to treat. Stepwise initial assessment includes semi‐structured interviews, clinical ratings and self‐reports (both mandatory and optional; DERS is optional); all recorded with software on clinicians' computers. ED diagnoses are based on the Structured Eating Disorder Interview that demonstrates good reliability and validity (de Man Lapidoth & Birgegård, 2010). The ~45‐min assessment is performed by trained professionals at the third clinic visit. The Stepwise 1‐year assessment, similar to the initial assessment, is performed one year after the initial evaluation, within a 10‐week window (±5 weeks). Attrition between initial and 1‐year assessment occurs and can be due to both patient and clinician/treatment unit factors (drop‐out, time constraints, follow‐ups not encouraged). Stepwise attrition is around 40–64%, with a trend of higher attrition, the longer Stepwise has been running (Andersén & Birgegård, 2017; Ekeroth & Birgegård, 2014; Forsén Mantilla, Norring, & Birgegård, 2019).

### 2.2 Participants

**FIGURE 1:**
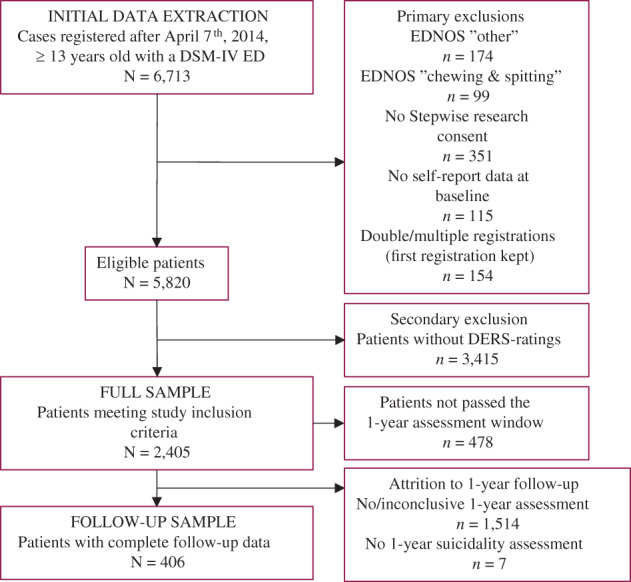
Flow chart describing participant exclusions and attrition at various levels [Color figure can be viewed at wileyonlinelibrary.com]

Initial data included 6,713 potential cases ≥13 years of age with a DSM‐IV ED registered between April 7, 2014 (date when DERS was included in Stepwise), and October 16, 2019 (date of data extraction). Exclusions were: low prevalence ED not otherwise specified (NOS) categories (“other”, chewing & spitting), no consent to participate in research, no baseline self‐reports, and multiple registrations (first registration retained) resulting in 5,820 cases (Figure 1). DSM‐IV diagnoses were recoded post‐hoc into DSM‐5 diagnoses, as reported previously (Andersén & Birgegård, 2017; Monell et al., 2018). Excluding patients without DERS ratings left 2,405 patients in the full sample (41% of eligible patients within the study time frame). The full sample comprised 1,557 adults and 848 adolescents aged 13–72 years (*M* = 22.5, SD = 8.6; detailed sample description in Table 1); 482 with AN‐R (20%), 133 with AN‐BP (5.5%), 710 with BN (29.5%), 100 with BED (4.2%), 463 with atypical AN (AAN; 19.3%), and 517 with OSFED (21.5%). One‐year assessment, including assessment of diagnostic status and suicidality, was available for 406 patients (follow‐up sample; Figure 1). One patient died during the follow‐up period (unknown cause of death; by clinician report). When excluding patients who had not yet passed the 1‐year follow‐up window, attrition rate was 78.9%.

**TABLE 1:** Sample description, clinical variables and suicidality at initial registration, and 1‐year follow‐up

																Diagnostic comparison	
		All EDs		AN‐R		AN‐BP		BN		BED		AAN		OSFED		*F*/*χ* ^2^	*p*
**Initial registration**																	
		(*N* = 2,405)		(*n* = 482)		(*n* = 133)		(*n* = 710)		(*n* = 100)		(*n* = 463)		(*n* = 517)			
*Mean*, *SD*																	
Age		22.46	8.55	18.50	6.43	20.95	6.70	25.57	8.64	31.83	11.17	20.20	7.46	22.49	8.00	82.73	<.001
BMI		21.65	6.09	16.41	1.74	17.01	2.03	25.06	5.90	34.35	9.55	20.27	3.18	21.83	3.74	416.77	<.001
ED duration		7.29	8.23	3.78	5.52	6.14	6.37	10.30	8.58	16.05	12.11	5.06	6.91	7.00	7.80	77.37	<.001
EDE‐Q Global score		3.78	1.31	3.12	1.56	3.90	1.39	4.14	1.01	3.58	.90	3.69	1.38	3.99	1.15	43.33	<.001
DERS non‐acceptance		16.15	6.33	14.95	6.06	17.02	6.57	16.55	6.23	16.16	6.26	16.03	6.39	16.62	6.49	5.20	<.001
DERS goals		16.73	5.35	16.26	5.46	17.09	5.03	17.24	5.14	17.16	4.90	16.41	5.54	16.57	5.50	2.71	.019
DERS impulse		14.89	6.35	14.03	6.18	15.54	6.46	15.65	6.35	15.49	6.18	14.71	6.56	14.51	6.21	4.71	<.001
DERS awareness		18.61	5.16	18.41	5.18	19.71	4.92	18.71	5.12	17.83	4.88	18.45	5.27	18.68	5.21	1.97	.080
DERS strategies		21.79	7.83	20.85	8.01	23.35	8.22	22.31	7.58	21.35	7.08	21.63	7.99	21.76	7.79	3.18	.007
DERS clarity		14.29	4.65	14.15	4.82	15.44	4.69	14.35	4.56	13.15	4.17	14.35	4.58	14.22	4.73	2.98	.011
DERS total score		102.46	26.75	98.65	26.79	108.14	26.07	104.81	26.26	101.14	23.60	101.58	27.74	102.36	26.78	4.43	.001
*N*, *%*																	
Gender (female)		2,303	95.8%	465	96.5%	130	97.7%	681	95.9%	96	96%	426	92%	505	97.7%	22.61	<.001
Depression	SCID‐I	452	34%	51	34%	23	33%	185	34%	28	33%	71	35%	94	32%	.48	.993
	Mini‐kid	310	46%	93	43%	24	59%	51	59%	3	50%	64	36%	75	51%	18.67	.002
Lifetime SA	No	2,003	83%	437	91%	98	74%	565	80%	74	74%	407	88%	421	82%	48.01	<.001
(valid N = 2,398)	Any	395	17%	44	9%	35	26%	139	20%	26	26%	56	12%	96	18%		
SI (valid N = 2,399)	No	1,475	61%	303	63%	70	53%	442	62%	63	63%	288	63%	309	60%	5.20	.392
	Any	924	39%	178	37%	61	47%	267	38%	37	37%	173	37%	208	40%		
**Follow‐up**																	
		(*N* = 406)		(*n* = 105)		(*n* = 22)		(*n* = 110)		(*n* = 18)		(*n* = 82)		(*n* = 69)			
*Mean*, *SD*																	
BMI		22.02	5.44	18.62	2.01	18.82	1.73	24.64	6.24	34.96	4.87	20.95	3.10	21.92	3.34	29.33	<.001
EDE‐Q Global score		2.11	1.60	1.65	1.49	2.57	1.95	2.29	1.58	2.21	1.26	1.97	1.70	2.51	1.48	1.93	.091
*N*,*%*																	
Gender (female)		391	96.3%	102	97.1%	22	100%	103	93.6%	18	100%	78	95.1%	68	98.6%	5.24	.387
No ED		227	56%	58	55%	12	55%	64	58%	15	83%	45	55%	33	48%	7.62	.178
SA follow‐up	No	390	96%	104	99%	21	95%	106	96%	18	100%	79	96%	62	90%	10.30	.067
	Any	16	4%	1	1%	1	5%	4	4%			3	4%	7	10%		
SI follow‐up	No	291	72%	77	73%	12	55%	80	73%	12	67%	60	73%	50	72%	3.72	.591
	Any	115	28%	28	27%	10	45%	20	27%	6	33%	22	27%	19	28%		

Abbreviations: AAN, atypical anorexia nervosa; AN‐BP, AN binge‐eating/purging type; AN‐R, AN restricting type; BED, binge‐eating disorder; BMI, body mass index; BN, bulimia nervosa; ED, eating disorder; EDE‐Q, Eating Disorder Examination Questionnaire; Depression, current depressive episode; DERS, Difficulties in Emotion Regulation Scale; Mini‐Kid, Mini International Neuropsychiatric Interview for Children and Adolescents; OSFED, other specific feeding and EDs; SA, occurrence of lifetime suicide attempts; SA follow‐up, occurrence of suicide attempts last 12 months; SCID‐I, Structured Clinical Interview for DSM‐IV axis I; SI, occurrence of suicidal ideation during the last three months; SI follow‐up, occurrence of suicidal ideation the last three months.

### 2.3 Measures

*The Structured Clinical Interview for DSM‐IV axis I* (SCID‐I) (First, Spitzer, Gibbon, & Williams, 2002) and *the Mini International Neuropsychiatric Interview for Children and Adolescents* (M.I.N.I. Kid) (Sheehan et al., 1998) assessed psychiatric comorbidity, for patients ≥18 and < 18 years, respectively. Current major depressive episode at baseline was dichotomized into 0 (no current depressive episode) or 1 (current depressive episode). The SCID‐I is mandatory, and the MINI‐Kid is optional. Depression data were available for 2021 participants (84% of the total sample; 86% of adults, and 80% of adolescents). Thus, sample sizes for analyses including covariates are smaller.

*Suicidality* at initial assessment and 1‐year follow‐up was extracted from the Riksät National Quality Registry for ED (embedded within Stepwise). Lifetime/follow‐up suicide attempts were recorded using the question “Has the patient ever (initial registration) / since [date for initial registration] (1‐year assessment) attempted suicide?” with fixed responses “Never”, “1–2 times”, and “more than 3 times”. Suicidal ideation was recorded using the question “How often during the past three months has the patient had suicidal thoughts, intentions or plans?” with the responses “Never”, “Occasionally”, and “Every week or more”. Suicidal ideation and suicide attempts at both time‐points were dichotomized such that “Never” was coded as 0 (=no suicidal ideation/suicide attempts), and alternatives “1–2 times” and “3 or more” for suicide attempts and “Occasionally” and “Every week or more” for suicidal ideation were combined into 1 (=any suicidal ideation/suicide attempt). Although mandatory, clinicians can note if sufficient information is lacking. At initial registration, small numbers of patients lacked information on suicidality (<1%); as no patient lacked information in both variables, all remained in the full sample. At follow‐up, all patients had information on both suicidality outcomes.

*The Eating Disorder Examination Questionnaire* (EDE‐Q) (Fairburn & Beglin, 1994), version 4.0, is a self‐report questionnaire assessing ED symptoms over the past 28 or 14 days (adult and adolescent version, respectively) (Carter, Stewart, & Fairburn, 2001) by 36 items rated 0–6. The EDE‐Q provides a mean Global Score and four subscales (restraint, eating concern, shape concern, and weight concern) with higher scores indicating greater pathology. The Swedish adult and adolescent versions have satisfactory validity and reliability (Forsén Mantilla, Birgegård, & Clinton, 2017; Welch, Birgegård, Parling, & Ghaderi, 2011). Internal consistency in this sample was excellent for the Global Score (mean Cronbach's *α* = .94). EDE‐Q is mandatory in Stepwise; all participants in the full sample had complete EDE‐Q ratings (missing item‐level data is technically prohibited in Stepwise).

*The DERS* (Gratz & Roemer, 2004) assesses emotion dysregulation with 36 items rated 1–5 (almost never – almost always), yielding six subscales and one Total scale (range 36–180).The subscales are: non‐acceptance (non‐accepting attitude towards emotional distress; range 6–36), goals (difficulties engaging in goal‐directed behaviors when upset; range 6–30), impulse (difficulties to control one's behavior when upset; range 6–36), awareness (inability to pay attention and be conscious about one's emotional responses; range 6–36), strategies (limited access to functional emotion regulation strategies when upset; range 6–42), and clarity (lack of emotional understanding and clarity; range 6–30). Higher scores indicate greater difficulty with emotion regulation. The Swedish DERS shows adequate psychometric properties in adults and adolescents with EDs (Monell, Bjureberg, Nordgren, Hesser, & Birgegård, 2020; Nordgren, Monell, Birgegård, Bjureberg, & Hesser, 2019). Internal consistency in this sample was excellent for goals and impulse (mean Cronbach αs = .90 and .91, respectively), and good for the remaining four subscales (αs = .81–.90).

### 2.4 Statistical analyses

Differences between ED diagnostic subgroups on suicidality, DERS, and covariates were explored in the full and follow‐up samples with *χ* ^2^–tests for categorical and analysis of variance (ANOVA) for continuous variables. Associations between initial DERS and suicidality at initial registration (aim 1 and 2) and 1‐year follow‐up (aim 3) were examined using logistic regression. Several regression models were examined for each suicidality measure. To correct for multiple comparisons, the Bonferroni correction method was applied within families of tests (family defined as each aim).

*Aim 1*: Considering the full sample (i.e., all EDs at initial registration), DERS total score was examined as a predictor of lifetime suicide attempts and recent suicidal ideation at initial registration (Main analyses; Model 1, unadjusted). Then, all DERS subscales were entered simultaneously as predictors of lifetime suicide attempts and recent suicidal ideation, respectively (Secondary analyses; Model 2, unadjusted).

*Aim 2*: Model 2 was repeated in all ED diagnostic subgroups separately at initial registration (Exploratory analyses).

*Aim 3*: For the follow‐up analyses, initial DERS total score was examined as a predictor of suicide attempts and suicidal ideation assessed at 1‐year follow‐up (Main analyses; Model 1, unadjusted) in the subset of participants with complete follow‐up data (i.e., the follow‐up sample). Then, initial DERS subscales were examined as predictors for follow‐up suicide attempts and suicidal ideation (Secondary analyses; Model 2, unadjusted).

All models were re‐fitted including age at initial registration, ED duration, EDE‐Q Global Score, and depression status as covariates (Models 1 and 2, adjusted); 1‐year models additionally included initial suicidality (suicide attempts and suicidal ideation assessed at initial registration). Statistical analyses were performed using the Statistical Package for the Social Sciences for Mac (SPSS‐22/24).

### 2.5 Sample representativeness

Sample representativeness was examined by comparing eligible patients with and without DERS at initial registration (Table S1), and participants in the full sample with and without follow‐up assessment (Table S2). Phi‐coefficients (Φ) for *χ* ^*2*^ with categorical variables and Cohen's *d* for *t*‐tests with continuous variables (*d* > .20/Φ > .10 considered small, *d* > .50/Φ > .30 considered medium, *d* > .80/Φ > .50 large effects) were computed for significant results. Statistically significant differences (*p* < .05) with at least small effect sizes were considered meaningful. No meaningful differences between patients with and without DERS emerged (Table S1) nor between adults with and without complete follow‐up assessments (Table S2). Adolescents with and without complete follow‐up differed in diagnostic distribution (mostly AN‐R and OSFED), past year suicide attempt, BMI, age, and lower DERS awareness and clarity (Table S2).

## 3 RESULTS

### 3.1 Sample characteristics

Sample description at initial registration and follow‐up is shown in Table 1. At initial registration, 17% of participants reported at least one lifetime suicide attempt (the lowest prevalence in AN‐R, and highest in AN‐BP and BED). Suicidal ideation was endorsed by 39% of participants with similar prevalence across diagnostic subgroups. Sixty‐three percent of those reporting suicide attempts also reported suicidal ideation (while 31.7% with suicidal ideation also had attempted suicide). Significant differences by diagnostic subgroup emerged in all DERS subscales except awareness. The typical pattern was AN‐R scoring lowest (except in clarity, where BED scored lowest); and AN‐BP or BN scoring highest.

At 1‐year follow‐up, 4% in the follow‐up sample had attempted suicide during the prior year, and 28% endorsed suicidal ideation. No significant differences in follow‐up suicidality emerged by initial diagnostic group.

### 3.2 Associations between emotion dysregulation and suicidality at initial registration

DERS total score was significantly associated with lifetime suicide attempts in the full sample, with a one‐point increase in the total score indicating 1.6% higher odds of life‐time suicide attempts (Table 2; Model 1). The association remained significant when accounting for covariates. DERS total score was also significantly associated with suicidal ideation, with a one‐point increase in the total score indicating 2.8% higher odds of recent suicidal ideation. Similar results emerged when adjusting for covariates. When entering all DERS subscales simultaneously (Table 2; Model 2), a one‐point increase in strategies indicated 4.1% increased odds of prior suicide attempts. Further, strategies and awareness were uniquely associated with suicidal ideation; one‐point increases indicated 5.5% and 9.6% increased odds of recent suicidal ideation, respectively. When accounting for covariates, associations remained significant (all covariate values in Table S3).

**TABLE 2:** Suicidality at initial registration: results of logistic regression analysis. Bonferroni corrected significance level: *p* < .0125

		Unadjusted			Adjusted		
Outcome	Predictor	*p*	OR	95% CI	*p*	OR	95% CI
**Model 1**							
SA	DERS total score	**<.001**	1.016	1.012–1.020	**<.001**	1.011	1.006–1.016
SI	DERS total score	**<.001**	1.028	1.024–1.032	**<.001**	1.019	1.015–1.024
**Model 2**							
SA	DERS non‐acceptance	.368	1.010	0.989–1.032	.403	0.990	0.966–1‐014
	DERS goals	.404	1.013	0.982–1.045	.657	1.008	0.973–1.044
	DERS impulse	.510	1.008	0.984–1.034	.293	1.015	0.987–1.044
	DERS awareness	.036	1.028	1.002–1.054	.131	1.022	0.994–1.052
	DERS strategies	**.001**	1.041	1.017–1.066	**.002**	1.043	1.015–1.071
	DERS clarity	.206	0.980	0.949–1.011	.089	0.969	0.935–1.005
SI	DERS non‐acceptance	.488	0.994	0.977–1.011	.391	0.991	0.972–1.011
	DERS goals	.724	0.996	0.971–1.020	.256	0.984	0.958–1.012
	DERS impulse	.118	1.016	0.996–1.037	.138	1.017	0.995–1.040
	DERS awareness	**<.001**	1.055	1.034–1.077	**.002**	1.036	1.103–1.060
	DERS strategies	**<.001**	1.096	1.075–1.117	**<.001**	1.077	1.054–1.100
	DERS clarity	.124	0.980	0.955–1.006	.224	0.982	0.955–1.011

*Note:* Model 1–2 run in the full sample (*N* = 2,398 for SA; 2,399 for SI).

Abbreviations: DERS, Difficulties in Emotion Regulation Scale; SA, lifetime suicide attempts (0 = no; 1 = yes); SI, suicidal ideation during the last three months (0 = no; 1 = yes).

No DERS subscale was significantly associated with lifetime suicide attempts in any ED diagnostic subgroup (Table 3; Model 2). Strategies was associated with suicidal ideation in all subgroups except BED; a one‐point increase in strategies indicated increased odds of recent suicidal ideation (AN‐R: 10.2%; AN‐BP: 13.9%; BN: 10.4%; AAN: 6.9%; OSFED: 10.1%). In AAN, a one‐point increase in awareness indicated 7.6% higher odds of recent suicidal ideation. When adjusting for covariates, strategies remained significant in AN‐R, BN, and OSFED; no DERS scale retained significance in AN‐BP and AAN (Table 4; Model 2).

**TABLE 3:** DERS subscales and suicide attempts at initial registration in each ED diagnostic subgroup. Bonferroni corrected significance level: *p* < .0042

			Unadjusted				Adjusted			
Model 2	Valid *n*	DERS predictor	Wald	*p*	OR	95% CI	Wald	*p*	OR	95% CI
AN‐R	481	Non‐acceptance	0.47	.493	0.977	0.914–1.044	0.05	.823	0.991	0.917–1.071
		Goals	0.37	.545	1.026	0.943–1.117	0.26	.607	1.027	0.927–1.138
		Impulse	0.08	.774	1.010	0.945–1.080	0.06	.806	0.990	0.911–1.075
		Awareness	0.94	.332	1.036	0.964–1.114	1.71	.191	1.058	0.972–1.152
		Strategies	3.25	.071	1.057	0.995–1.123	3.55	.059	1.071	0.997–1.150
		Clarity	1.98	.160	1.063	0.976–1.156	0.50	.478	1.038	0.937–1‐150
AN‐BP	133	Non‐acceptance	0.14	.708	1.014	0.943–1.091	2.20	.138	0.927	0.839–1.025
		Goals	0.12	.733	0.979	0.866–1.107	<0.01	.988	1.001	0.857–1.170
		Impulse	1.15	.284	0.950	0.866–1.043	1.26	.262	0.935	0.831–1.052
		Awareness	1.04	.309	0.953	0.868–1.046	0.08	.773	1.018	0.900–1.153
		Strategies	5.04	.025	1.112	1.014–1.219	4.58	.032	1.154	1.012–1.317
		Clarity	0.07	.797	1.015	0.905–1.138	0.11	.743	0.972	0.823–1.149
BN	704	Non‐acceptance	0.81	.368	1.018	0.980–1.057	0.14	.709	1.008	0.967–1.050
		Goals	1.38	.241	1.034	0.978–1.094	0.31	.579	1.017	0.958–1.080
		Impulse	0.01	.946	0.999	0.956–1.042	0.54	.463	1.018	0.971–1.066
		Awareness	0.32	.570	1.013	0.969–1.059	0.06	.800	1.006	0.960–1.055
		Strategies	4.14	.042	1.047	1.002–1.095	3.33	.068	1.045	0.997–1.096
		Clarity	4.86	.027	0.936	0.883–0.993	5.47	.019	0.928	0.872–0.988
BED	100	Non‐acceptance	0.14	.711	1.018	0.925–1.121	<0.01	.995	1.000	0.901–1.109
		Goals	0.44	.508	0.957	0.842–1.089	0.02	.883	0.988	0.842–1.159
		Impulse	0.29	.589	0.971	0.871–1.082	0.46	.497	0.957	0.842–1.087
		Awareness	0.04	.834	0.987	0.878–1.111	0.37	.545	0.958	0.832–1.102
		Strategies	0.26	.611	1.026	0.930–1.131	0.09	.770	1.019	0.899–1.155
		Clarity	0.25	.620	0.964	0.835–1.113	0.05	.827	0.983	0.839–1.151
AAN	463	Non‐acceptance	4.55	.033	1.061	1.005–1.121	0.80	.372	1.030	0.965–1.099
		Goals	0.02	.878	0.994	0.914–1.079	0.23	.632	0.977	0.888–1.075
		Impulse	1.55	.213	1.042	0.977–1.111	1.47	.225	1.047	0.972–1.128
		Awareness	1.21	.272	1.038	0.972–1.108	0.25	.620	1.020	0.945–1.100
		Strategies	0.01	.943	0.998	0.939–1.060	0.01	.918	0.996	0.927–1.070
		Clarity	3.69	.055	1.090	0.998–1.190	1.90	.168	1.073	0.971–1.187
OSFED	517	Non‐acceptance	2.52	.113	0.964	0.922–1.009	4.53	.033	0.946	0.899–0.996
		Goals	0.13	.723	0.988	0.926–1.055	0.03	.860	0.993	0.922–1.070
		Impulse	0.73	.393	1.024	0.970–1.080	2.41	.121	1.049	0.988–1.114
		Awareness	7.81	.005	1.080	1.023–1.139	5.53	.019	1.076	1.012–1.144
		Strategies	3.72	.054	1.051	0.999–1.105	2.41	.121	1.048	0.988–1.113
		Clarity	3.00	.083	0.945	0.886–1.008	3.90	.048	0.926	0.859–0.999

*Note:* Lifetime suicide attempts (0 = no; 1 = yes).

Abbreviations: AAN, atypical anorexia nervosa; AN‐BP, anorexia nervosa binge‐eating/purging type; AN‐R, anorexia nervosa restricting type; BN, bulimia nervosa; BED, binge‐eating disorder; DERS, Difficulties in Emotion Regulation Scale; OSFED, other specified feeding and eating disorders.

**TABLE 4:** DERS subscales and recent suicidal ideation at initial registration in each ED diagnostic subgroup. Bonferroni corrected significance level: *p* < .0042

			Unadjusted				Adjusted			
Model 2	Valid *n*	DERS predictor	Wald	*p*	OR	95% CI	Wald	*p*	OR	95% CI
AN‐R	481	Non‐acceptance	0.01	.933	1.002	0.960–1.045	0.15	.702	1.010	0.960–1.063
		Goals	0.19	.666	1.012	0.960–1.066	0.01	.934	1.003	0.941–1‐069
		Impulse	<0.01	.969	1.001	0.957–1.047	0.02	.897	0.996	0.942–1.054
		Awareness	7.79	.005	1.068	1.020–1.118	5.30	.021	1.067	1.010–1.129
		Strategies	22.20	**<.001**	1.102	1.058–1.148	11.90	**.001**	1.087	1.037–1.140
		Clarity	1.53	.217	0.965	0.913–1.021	1.46	.228	0.958	0.895–1.027
AN‐BP	131	Non‐acceptance	0.50	.480	1.025	0.958–1.096	<0.01	.985	0.999	0.915–1.091
		Goals	0.07	.789	0.984	0.877–1.104	0.03	.857	1.012	0.886–1.157
		Impulse	<0.01	.996	1.000	0.917–1.090	0.03	.865	1.009	0.907–1.124
		Awareness	3.83	.050	1.099	1.000–1.208	4.02	.045	1.124	1.003–1.260
		Strategies	8.63	.**003**	1.139	1.044–1.242	3.45	.063	1.109	0.994–1.237
		Clarity	4.94	.026	0.879	0.784–0.985	2.59	.108	0.890	0.772–1.026
BN	709	Non‐acceptance	0.01	.915	0.998	0.966–1.032	<0.01	.977	1.001	0.966–1.036
		Goals	0.30	.583	0.986	0.940–1.036	0.81	.368	0.977	0.928–1.028
		Impulse	2.79	.095	1.033	0.994–1.072	1.68	.195	1.027	0.987–1.068
		Awareness	5.32	.021	1.047	1.007–1.088	3.61	.058	1.040	0.999–1.083
		Strategies	24.41	**<.001**	1.104	1.061–1.147	16.96	**<.001**	1.090	1.046–1.136
		Clarity	2.91	.088	0.958	0.911–1.006	3.07	.080	0.954	0.906–1.006
BED	100	Non‐acceptance	0.01	.932	0.996	0.911–1.089	0.07	.792	0.987	0.894–1.089
		Goals	3.68	.055	0.879	0.770–1.003	2.72	.099	0.880	0.756–1.024
		Impulse	0.93	.334	1.051	0.950–1.164	1.36	.243	1.069	0.956–1.195
		Awareness	<0.01	.990	1.001	0.899–1.113	0.19	.663	0.974	0.865–1.096
		Strategies	2.36	.125	1.077	0.980–1.184	0.75	.387	1.050	0.940–1.174
		Clarity	0.01	.939	1.005	0.881–1.147	0.06	.805	1.018	0.884–1.173
AAN	461	Non‐acceptance	0.54	.463	1.015	0.975–1.056	0.11	.740	1.008	0.962–1.055
		Goals	0.01	.941	1.002	0.948–1.060	0.56	.456	0.976	0.916–1.040
		Impulse	0.83	.363	1.022	0.975–1.072	0.56	.454	1.021	0.968–1.077
		Awareness	9.23	**.002**	1.076	1.026–1.127	2.63	.105	1.045	0.991–1.101
		Strategies	8.97	.**003**	1.069	1.023–1.116	3.62	.057	1.049	0.999–1.102
		Clarity	0.03	.869	0.995	0.935–1.058	0.03	.869	1.006	0.939–1.077
OSFED	517	Non‐acceptance	5.24	.022	0.957	0.921–0.994	5.12	.024	0.952	0.913–0.993
		Goals	0.01	.934	1.002	0.950–1.057	0.01	.924	0.997	0.939–1.058
		Impulse	0.16	.686	1.009	0.965–1.055	0.37	.545	1.015	0.966–1.067
		Awareness	1.73	.189	1.030	0.985–1.077	0.03	.859	0.995	0.947–1.047
		Strategies	19.62	**<.001**	1.101	1.055–1.149	8.77	.**003**	1.077	1.025–1.131
		Clarity	1.17	.279	1.030	0.976–1.088	1.05	.305	1.033	0.971–1.099

*Note:* Suicidal ideation during the last three months (0 = no; 1 = yes).

Abbreviations: AAN, atypical anorexia nervosa; AN‐BP, anorexia nervosa binge‐eating/purging type; AN‐R, anorexia nervosa restricting type; BED, binge‐eating disorder; BN, bulimia nervosa; DERS, Difficulties in Emotion Regulation Scale; OSFED, other specified feeding and eating disorders.

### 3.3 Associations between initial emotion dysregulation and suicidality at 1‐year follow‐up

Initial DERS total score was a significant predictor of follow‐up suicide attempts, with a one‐point increase in initial total score indicating 3.3% higher odds of at least one attempt during the following year (Table 5; Model 1). This association did not remain after accounting for covariates; occurrence of lifetime suicide attempts was the only significant predictor of follow‐up attempts (OR 10.46, *p* = .001). Initial DERS total score was also a significant predictor of follow‐up suicidal ideation, with a one‐point increase in the total score indicating 2% higher risk of suicidal ideation at follow‐up. After adjusting for covariates, this association did not remain significant; only initial suicidal ideation was significantly associated with suicidal ideation at follow‐up (OR 6.65, *p* < .001).

**TABLE 5:** Suicidality at 1‐year follow‐up: results of logistic regression analysis. Bonferroni corrected significance level: *p* < .0125

Outcome	Predictor	Unadjusted			Adjusted		
		*p*	OR	95% CI	*p*	OR	95% CI
*Model 1*							
SA follow‐up	DERS total score	**.003**	1.033	1.011–1.055	.950	1.001	0.972–1.031
SI follow‐up	DERS total score	**<.001**	1.020	1.011–1.029	.506	1.004	0.992–1.017
*Model 2*							
SA follow‐up	DERS non‐acceptance	.987	0.999	0.904–1.105	.739	0.979	0.863–1.110
	DERS goals	.408	0.932	0.790–1.101	.209	0.879	0.720–1.075
	DERS impulse	.057	1.128	0.996–1.277	.258	1.091	0.938–1.269
	DERS awareness	.290	1.071	0.943–1.216	.296	1.082	0.933–1.255
	DERS strategies	.422	1.043	0.941–1.155	.715	1.025	0.898–1.170
	DERS clarity	.933	1.007	0.865–1.171	.566	0.948	0.790–1.138
SI follow‐up	DERS nonacceptance	.555	1.013	0.971–1.057	.351	1.025	0.973–1.080
	DERS goals	.370	1.029	0.966–1.096	.795	1.010	0.937–1.088
	DERS impulse	.947	0.998	0.950–1.050	.108	0.952	0.897–1.011
	DERS awareness	.339	1.026	0.973–1.083	.705	0.988	0.926–1.053
	DERS strategies	**.009**	1.060	1.015–1.108	.259	1.032	0.977–1.089
	DERS clarity	.304	0.966	0.905–1.032	.818	1.009	0.933–1.092

*Note:* Model 1–2 run in the follow‐up sample (*N* = 406). SA follow‐up: suicide attempts last 12 months (0 = no; 1 = yes); SI follow‐up: suicidal ideation the last three months (0 = no; 1 = yes).

Abbreviation: DERS: Difficulties in Emotion Regulation Scale.

When entering all initial DERS subscales simultaneously, no subscale predicted follow‐up suicide attempts (Table 5; Model 2). When including covariates, only occurrence of lifetime suicide attempts was a significant predictor of follow‐up attempts (OR 10.28, *p* = .001). One‐point increase in initial strategies indicated 6% increased risk of occurrence of follow‐up suicidal ideation. When accounting for covariates, only initial suicidal ideation was a significant predictor of follow‐up suicidal ideation (OR 6.86, *p* < .001; all covariate values in Table S4).

## 4 DISCUSSION

Emotion dysregulation could be a potential mechanism contributing to suicidality in EDs. In this large sample of patients with EDs, higher overall emotion dysregulation was associated with increased odds of both lifetime suicide attempts and recent suicidal ideation at initial registration. Further, higher perceived lack of adaptive emotion regulation strategies was uniquely associated with both suicidality outcomes, and higher difficulties in emotional awareness was associated with suicidal ideation. Previous research on emotion dysregulation and suicidality is limited to comparisons between patients with or without lifetime suicide attempts (Gomez‐Exposito et al., 2016; Pisetsky, Haynos, et al., 2017; Smith, Forrest, et al., 2018). Our findings partially corroborate those of Smith et al., highlighting lack of adaptive emotion regulation strategies; however, as our methodology enabled identification of unique associations with suicidality, results are not directly comparable. In general, suicidality prevalence in this sample was in line with previous findings (Franko & Keel, 2006; Milos et al., 2004; Smith, Zuromski, et al., 2018; Swanson et al., 2011; Udo et al., 2019).

Rather than ED diagnosis‐specific associations between aspects of emotion dysregulation and suicidality, our results suggest more of a transdiagnostic pattern of perceived lack of strategies influencing suicidal ideation (except in BED). No emotion dysregulation measure was uniquely associated with suicide attempts in any diagnostic group, despite diagnostic differences in suicide attempts prevalence (i.e., AN‐R lowest, AN‐BP and BED highest). Of note, DERS‐defined emotion dysregulation and suicidal ideation both refer to current cognitive‐emotional processes at initial registration, in contrast to self‐report lifetime suicide attempts referring to behaviors at any previous time point. Thus, associations with suicidal ideation were likely easier to detect. Moreover, the small sample size in each ED group, and the conservative correction for multiple comparisons may have limited the power to detect diagnosis‐specific patterns of associations between emotion dysregulation domains and suicidality, especially in BED which was underrepresented.

Emotion dysregulation independently contributed to suicidality, beyond the effect of ED psychopathology and current depression. Our results extend the understanding of suicidality in EDs; emotion dysregulation specifically impacted on suicidality even when controlling for relevant clinical variables. ED diagnoses and symptoms clearly impact on suicidality, with strong evidence of associations between suicidality and bulimic spectrum EDs and compensatory behaviors (Ahn, Lee, & Jung, 2019). Up to 45% of patients with EDs with a history of suicidality report having attempted suicide before ED onset, indicating that shared factors underlying ED and suicidality should be considered (Udo et al., 2019). Although present results confirm higher prevalence of suicidality in AN‐BP, BED, and BN, the specific features of eating psychopathology do not appear to influence the association between emotion dysregulation and suicidality. Contrary to prior research (Ahn et al., 2019; Bulik et al., 2008; Pisetsky et al., 2013; Pisetsky et al., 2015; Udo et al., 2019), depression was not associated with lifetime suicide attempts in the multivariate analyses, although it was associated with recent suicidal ideation. This is surprising, given that depression is reported 4.32–15.06‐fold times more frequently in patients with EDs who report a history of suicide attempts than in those without (Udo et al., 2019). Additionally, previous research on BN indicated depression as the salient factor for lifetime suicide attempts when examined with emotion dysregulation‐related personality factors (Pisetsky et al., 2015). Our results, in contrast, revealed a marked association between emotion dysregulation and suicidality that was both stronger than, and independent of, depression. This lack of agreement could reflect different assessment of emotion related concepts (i.e., personality facets instead of DERS); furthermore, associations might have emerged with a continuous measure of depression instead of the dichotomous depression variable used here.

Emotion dysregulation at initial registration longitudinally predicted future suicidality, indicating emotion dysregulation as a risk trait. Although the small sample, higher initial overall emotion dysregulation was associated with increased risk of both suicidality outcomes in the year following initial assessment. Only one prior study has shown preliminary evidence of the role of emotion dysregulation in predicting future suicidality in EDs. Franko and colleagues explored a wide range of clinical variables at initial assessment as predictors of suicide attempts over the following 9 years in individuals with AN and BN; those with BN exhibiting greater impairment in identifying internal states (i.e., similar to DERS awareness and/or clarity) had a greater risk of suicide attempts (Franko et al., 2004). Although difficulties in identifying internal states cannot be considered as a proxy of the entire emotion dysregulation construct, the study by Franko et al. was a unique contribution to understanding longitudinal correlates of suicidality in EDs. The inclusion of initial suicidality in the models diminished the association of emotion dysregulation with future suicidality. This is consistent with prior studies identifying prior suicidality as the strongest predictor of future suicidality (Cavanagh, Carson, Sharpe, & Lawrie, 2003; Franko et al., 2004; Harris & Barraclough, 1997). Its replicated strength as a predictor should not detract from exploring the role of emotion dysregulation in being a clinical warning sign for future suicide risk.

The associations between emotion dysregulation and past, and follow‐up suicidality may relate to the interpersonal theory of suicide (ITPS) (Joiner, 2005). The ITPS, supported in several psychiatric populations including EDs (Pisetsky, Crow, & Peterson, 2017; Van Orden et al., 2010), posits that thwarted belongingness and perceived burdensomeness (inducing hopelessness and negative emotions) may increase suicidal ideation, which in turn enables suicide attempts, if coupled with an acquired capability for suicide (reduced fear of death, increased pain tolerance) and repeated exposure to fearful and/or painful experiences. Emotion dysregulation may play a role at various stages in this model. As emotions provide meaning to experiences, reduced emotional awareness may contribute to suicidal ideation by alienating the individual from oneself and others, thereby increasing depression and hopelessness (De Berardis et al., 2017). Lacking adaptive strategies to regulate negative emotions may trigger both suicidal ideation and suicidal behaviors, providing a sense of having the means of eventually escaping emotional pain (Brown, 2006). Therefore, lacking adaptive ways to regulate negative emotions may contribute to progressing from ideation to attempt. In this frame, prior suicide attempt indicates that the individual has already acquired the capability for suicide, which may explain the predictive power of prior attempts over other indicators. Symptoms such as purging and self‐starvation, along with other potentially self‐harming behaviors (i.e., potentially life‐threatening, requiring increased pain tolerance), may also infer greater risk for suicide attempts within ED populations. However, a concurrent examination of IPTS elements, ED symptoms, and emotion dysregulation, including other modeling approaches, would provide clearer guidance for detecting individuals at risk at various time points.

In order to prevent suicide in EDs, identification of therapeutically targetable traits associated with past and future suicide attempts is of considerable importance. Our results suggest that emotion dysregulation may represent such a trait that applies transdiagnostically. Although prior suicide attempt remains the most robust predictor of subsequent attempts, emotion dysregulation is both measurable and targetable therapeutically. Even though the mechanisms underlying the association between emotion dysregulation and suicidality in EDs are not fully understood (e.g., mediator, shared underlying psychological processes), both ED symptoms and suicidal behaviors are negatively reinforced by providing temporary relief from negative emotions—at the expense of more adaptive regulatory strategies (Skinner, Rojas, & Veilleux, 2017). Targeting emotion dysregulation may be beneficial in ED treatment for patients with and without past suicidality. A review of emotion dysregulation‐oriented interventions for various psychiatric disorders (e.g., ED, depression, anxiety, borderline personality disorder) revealed that improving emotion regulation skills was associated with decreases in both the specific pathology being targeted as well as comorbid psychopathology (Sloan et al., 2017). Using such approaches to address ED symptoms may also serve to reduce suicidality. Emotion regulation‐focused therapies such as Dialectical Behavior Therapy, Emotion Acceptance Behavior Therapy, and Integrative Cognitive‐Affective Therapy have been developed or adapted for EDs (Berg & Wonderlich, 2013). Whether they reduce both ED pathology and suicidality remains to be examined.

Study strengths include a large, ecologically valid sample at initial registration. The sample also included a wide range of DSM‐defined EDs, strengthening the representativeness of the results and enabling analyses of diagnostic subgroups. Several limitations should be considered. The DERS was optional, and clinicians' decisions to include DERS were not recorded. However, previous analysis of missing data showed that clinicians typically choose no optional measures (Monell et al., 2018), suggesting that rather than choosing measures based on patient characteristics, clinician/clinic variables (e.g., interest, time constraints, unit specific assessment routines) seemed most influential. Although no meaningful differences between patients with and without DERS emerged, unmeasured differences could have introduced bias. Even though depression assessment is mandatory, 16% lacked information on depression, meaning sample sizes for adjusted models were smaller. Moreover, in order to include adolescents, we had to dichotomize the depression variable, reducing statistical power. Similarly, suicidality variables were dichotomized, since the structure of the Riksät suicidality response options does not lend itself to ordinal quantification (i.e., never, occasionally, weekly for suicidal ideation; never, 1–2, ≥3 for suicide attempts), again reducing power. Further, we had limited information on the timing and seriousness of previous suicide attempts. In the follow‐up sample, only 4% and 28% reported suicide attempts and ideation, respectively; thus, analyses may have had too low power to detect a significant association between emotion dysregulation and suicidality beyond the effect of previous suicidality. Substantial attrition at one‐year follow‐up potentially threatened the representativeness of these analyses. However, multiple studies using the Stepwise registry reported small to negligible differences between patients with and without follow‐up data, indicating that factors related to treatment units rather than patient factors lead to attrition. We observed some minor differences between adolescents with and without follow‐up, indicating that our follow‐up results may be more generalizable to adolescents with AN‐R and those without past year suicide attempts (as these groups were more likely to have follow‐up data), and to those with slightly more difficulties in emotional clarity and awareness. We were unable to link our data to the Swedish Death Registry. Accordingly, other deaths could have occurred but not been recorded by the clinician. Lastly, as the sample comprised Swedish patients seeking active treatment, results may not generalize to nontreatment seeking groups or to more culturally diverse samples.

## 5 CONCLUSIONS

Suicidality in EDs is high, and robust and clinically relevant predictors of future attempts are needed. Emotion dysregulation was associated with both lifetime suicide attempts, recent ideation, and suicidality at 1‐year follow‐up in patients with a wide range of EDs, even when ED psychopathology and depression were accounted for. Results suggest that although suicidality differed across different EDs, emotion dysregulation may be a transdiagnostic trait influencing suicidality. Finally, these results encourage further longitudinal studies examining the specific contribution of emotion dysregulation to suicidality in EDs.

## CONFLICT OF INTEREST

Cynthia M. Bulik is a grant recipient from and a Scientific Advisory Board member for Shire, consultant for Idorsia and received royalties from Pearson. The other authors have no potential conflicts of interest to report.

## ROLE OF THE FUNDER/SPONSOR

The sponsors had no role in the design and conduct of the study; collection, management, analysis, and interpretation of the data; preparation, review, or approval of the manuscript; and decision to submit the manuscript for publication.

## Supporting information


**Table S1** Comparison between patients without and with DERS in relevant variables at initial registration (suicidality, sample characteristics, ED psychopathology, emotional symptoms). N for specific measures differs slightly so valid N is reported for each measure. CPRS‐S‐A is only available for adults; SDQ only for adolescents. Depression is based on SCID for adults and MINI‐Kid for adolescents. Categorical variables examined by χ^2^, continuous measures by independent samples *t*‐test. Effect sizes were considered small Φ > .10/ Cohen's *d* > .20, medium Φ > .30/ *d* > .50, and large Φ > .50/ *d* > .80. Comparisons were done in the whole sample (i.e., all ages; No DERS N = 3,415; DERS N = 2,405), and then separately for adults (No DERS N = 2,148; DERS N = 1,557) and adolescents (No DERS N = 1,267; DERS N = 848). Significant differences are in bold (all effect sizes were less than small).
**Table S2**. Comparison between patients with and without complete 1‐year follow‐up (FU; those without FU that technically could have such data, see manuscript) assessment in relevant variables at initial registration. N for specific measures differs slightly so valid N is reported for each measure. CPRS‐S‐A is only available for adults; SDQ only for adolescents. Depression is based on SCID for adults and MINI‐Kid for adolescents. Categorical variables examined by χ^2^, continuous measures by independent samples *t*‐test. Effect sizes were considered small Φ > .10/ Cohen's *d* > .20, medium Φ > .30/ *d* > .50, and large Φ > .50/ *d* > .80. Comparisons done in the whole sample (No FU N = 1,521; FU N = 406), then separately for adults (No FU N = 1,023; FU N = 237) and adolescents (No FU N = 498; DERS N = 169). Significant differences at level *p* < .05 and ≥ small effect sizes are highlighted.
**Table S3**. Suicidality at initial registration: results of logistic regression analysis. Bonferroni corrected significance level: *p* < .0125.
**Table S4**. Suicidality at 1 year follow‐up: results of logistic regression analysis. Bonferroni corrected significance level: *p* < .0125.

## Data Availability

The data belong to the Stepwise Registry and are not available for sharing.
