# Supplementary material for: Emotion dysregulation and suicidality in eating disorders
Source: Int J Eat Disord. 2020 Nov 18;54(3):313–25. doi: 10.1002/eat.23410 (PMC7984062; doi:10.1002/eat.23410)
Supplement: Supplementary file 1 — Table S1 Comparison between patients without and with DERS in relevant variables at initial registration (suicidality, sample characteristics, ED psychopathology, emotional symptoms). N for specific measures differs slightly so valid N is reported for each measure. CPRS‐S‐A is only available for adults; SDQ only for adolescents. Depression is based on SCID for adults and MINI‐Kid for adolescents. Categorical variables examined by χ2, continuous measures by independent samples t‐test. Effect sizes were considered small Φ > .10/ Cohen's d > .20, medium Φ > .30/ d > .50, and large Φ > .50/ d > .80. Comparisons were done in the whole sample (i.e., all ages; No DERS N = 3,415; DERS N = 2,405), and then separately for adults (No DERS N = 2,148; DERS N = 1,557) and adolescents (No DERS N = 1,267; DERS N = 848). Significant differences are in bold (all effect sizes were less than small). Table S2. Comparison between patients with and without complete 1‐year follow‐up (FU; those without FU that technically could have such data, see manuscript) assessment in relevant variables at initial registration. N for specific measures differs slightly so valid N is reported for each measure. CPRS‐S‐A is only available for adults; SDQ only for adolescents. Depression is based on SCID for adults and MINI‐Kid for adolescents. Categorical variables examined by χ2, continuous measures by independent samples t‐test. Effect sizes were considered small Φ > .10/ Cohen's d > .20, medium Φ > .30/ d > .50, and large Φ > .50/ d > .80. Comparisons done in the whole sample (No FU N = 1,521; FU N = 406), then separately for adults (No FU N = 1,023; FU N = 237) and adolescents (No FU N = 498; DERS N = 169). Significant differences at level p < .05 and ≥ small effect sizes are highlighted. Table S3. Suicidality at initial registration: results of logistic regression analysis. Bonferroni corrected significance level: p < .0125. Table S4. Suicidality at 1 year follow‐up: results of logistic regression ana [file EAT-54-313-s001.docx]

**SUPPORTING INFORMATION**

**Article: “Emotion dysregulation and suicidality in eating disorders”, by Rania, Monell, Sjölander, and Bulik**

| **Table S1.** Comparison between patients without and with DERS in relevant variables at initial registration (suicidality, sample characteristics, ED psychopathology, emotional symptoms). N for specific measures differs slightly so valid N is reported for each measure. CPRS-S-A is only available for adults; SDQ only for adolescents. Depression is based on SCID for adults and MINI-Kid for adolescents. Categorical variables examined by χ^2^, continuous measures by independent samples t-test. Effect sizes were considered small Φ>.10/ Cohen’s *d*>.20, medium Φ>.30/ *d*>.50, and large Φ>.50/ *d*>.80. Comparisons were done in the whole sample (i.e., all ages; No DERS N=3415; DERS N=2405), and then separately for adults (No DERS N=2148; DERS N=1557) and adolescents (No DERS N=1267; DERS N=848). Significant differences are in bold (all effect sizes were less than small). | | | | | | | | | | | | | | | | | | | | | |
| --- | --- | --- | --- | --- | --- | --- | --- | --- | --- | --- | --- | --- | --- | --- | --- | --- | --- | --- | --- | --- | --- |
|  | | All ages | | | | | |  |  |  | Adults | | |  |  |  | Adolescents | |  |  |  |
| N, % | No DERS | | | | | | DERS | χ^2^ | *p* | Φ | No DERS | | DERS | χ^2^ | *p* | Φ | No DERS | DERS | χ^2^ | *p* | Φ |
| SA | 474 (14%) | | | | | | 396 (16.5%) | 7.147 | **.008** | .035 | 398 (18.7%) | | 318 (20.5%) | 1.851 | .174 | .022 | 76 (6%) | 78 (9.2%) | 7.735 | **.005** | .061 |
| valid N | 3394 | | | | | | 2398 |  |  |  | 2132 | | 1554 |  |  |  | 1262 | 844 |  |  |  |
| SA recent | 137 (29.1%) | | | | | | 115 (29.1%) | .000 | .950 | <.001 | 94 (23.7%) | | 65 (20.5%) | 1.025 | .311 | -.038 | 43 (58.1%) | 50 (64.1%) | .575 | .448 | .061 |
| valid N | 471 | | | | | | 395 |  |  |  | 397 | | 317 |  |  |  | 74 | 78 |  |  |  |
| SI | 1204 (35.4%) | | | | | | 924 (38.5%) | 5.916 | **.015** | .032 | 714 (33.4%) | | 565 (36.4%) | 3.487 | .062 | .031 | 490 (38.8%) | 359 (42.5%) | 2.913 | .088 | .037 |
| valid N | 3402 | | | | | | 2399 |  |  |  | 2138 | | 1554 |  |  |  | 1264 | 845 |  |  |  |
| Depression | 1039 (38%) | | | | | | 762 (37.7%) | .057 | .811 | -.003 | 695 (37.6%) | | 452 (33.6%) | 5.382 | .020 | -.041 | 344 (39%) | 310 (45.9%) | 7.423 | **.006** | .069 |
| valid N | 2731 | | | | | | 2021 |  |  |  | 1850 | | 1346 |  |  |  | 881 | 675 |  |  |  |
| Males | 150 (4.4%) | | | | | | 102 (4.2%) | .078 | .780 | -.004 | 96 (4.5%) | | 72 (4.6%) | .050 | .823 | .004 | 54 (4.3%) | 30 (3.5%) | .699 | .403 | -.018 |
| valid N |  | | | | | |  |  |  |  | 2148 | | 1557 |  |  |  | 1267 | 848 |  |  |  |
| Age category distribution | | | |  | | | | 2.068 | .150 | .019 |  | |  |  |  |  |  |  |  |  |  |
| Adults | 2148 (62.9%) | | | | | | 1557 (64.7%) |  |  |  |  | |  |  |  |  |  |  |  |  |  |
| Adolescents | 1267 (37.1%) | | | | | | 848 (35.3%) |  |  |  |  | |  |  |  |  |  |  |  |  |  |
| Diagnostic distribution | | | |  | | | | 5.034 | .412 | .029 |  | |  | 7.128 | .211 | .044 |  |  | 1.183 | .947 | .024 |
| AN-R | 712 (20.8%) | | | | | | 482 (20%) |  |  |  | 283 (13.2%) | | 199 (12.8%) |  |  |  | 429 (33.9%) | 283 (33.4%) |  |  |  |
| AN-BP | 191 (5.6%) | | | | | | 133 (5.5%) |  |  |  | 119 (5.5%) | | 84 (5.4%) |  |  |  | 72 (5.7%) | 49 (5.8%) |  |  |  |
| BN | 1031 (30.2%) | | | | | | 710 (29.5%) |  |  |  | 889 (41.4%) | | 609 (39.1%) |  |  |  | 142 (11.2%) | 101 (11.9%) |  |  |  |
| BED | 162 (4.7%) | | | | | | 100 (4.2) |  |  |  | 152 (7.1%) | | 93 (6%) |  |  |  | 10 (.8%) | 7 (.8%) |  |  |  |
| AAN | 659 (19.3) | | | | | | 463 (19.3) |  |  |  | 296 (13.8%) | | 234 (15%) |  |  |  | 363 (28.7%) | 229 (27%) |  |  |  |
| OSFED | 660 (19.3%) | | | | | | 517 (21.5%) |  |  |  | 409 (19%) | | 338 (21.7%) |  |  |  | 251 (19.8%) | 179 (21.1%) |  |  |  |
|  | | All ages | | | | | |  |  |  | Adults | | |  |  |  | Adolescents | |  |  |  |
| *Mean, SD* | No DERS | | | | | DERS | | T | *p* | *d* | No DERS | | DERS | T | *p* | *d* | No DERS | DERS | T | *p* | *d* |
| Age | 22.39 (9.04) | | | | | 22.46 (8.55) | | -.320 | .749 |  | 26.75 (8.8) | | 25.45 (8.19) | 1.068 | .286 |  | 14.99 (1.37) | 15.15 (1.30) | -2.646 | **.008** | .120 |
| valid N | 3415 | | | | | 2405 | |  |  |  | 2148 | | 1557 |  |  |  | 1267 | 848 |  |  |  |
| ED-duration | 7.07 (8.61) | | | | | 7.29 (8.23) | | -.949 | .342 |  | 10.42 (9.30) | | 10.36 (8.78) | .180 | .857 |  | 1.41 (1.51) | 1.63 (1.67) | -3.249 | **.001** | .138 |
| valid N | 3412 | | | | | 2405 | |  |  |  | 2148 | | 1557 |  |  |  | 1267 | 848 |  |  |  |
| BMI | 21.24 (5.67) | | | | | 21.63 (6.12) | | -2.502 | **.012** | .066 | 22.55 (6.29) | | 23.15 (6.71) | -2.788 | **.005** | .092 | 19.02 (3.46) | 18.84 (3.40) | 1.173 | .241 |  |
| valid N | 3412 | | | | | 2405 | |  |  |  | 2148 | | 1557 |  |  |  | 1267 | 848 |  |  |  |
| EDE-Q Global | 3.74 (1.29) | | | | | 3.79 (1.31) | | -1.113 | .266 |  | 3.90 (1.56) | | 3.96 (1.14) | -1.367 | .172 |  | 3.46 (1.45) | 3.45 (1.53) | .152 | .880 |  |
| valid N | 3415 | | | | | 2405 | |  |  |  | 2148 | | 1557 |  |  |  | 1267 | 848 |  |  |  |
| CPRS-S-A depression | | |  | | |  | |  |  |  | 10.82 (4.71) | | 11.17 (4.57) | -2.217 | **.027** | .074 |  |  |  |  |  |
| valid N |  | | | | |  | |  |  |  | 2148 | | 1557 |  |  |  |  |  |  |  |  |
| CPRS-S-A anxiety | | |  | | |  | |  |  |  | 9.86 (4.25) | | 10.24 (4.29) | -2.581 | **.010** | .086 |  |  |  |  |  |
| valid N |  | | | | |  | |  |  |  | 2148 | | 1557 |  |  |  |  |  |  |  |  |
| SDQ emotional symptoms | | | | |  | | |  |  |  |  |  | |  |  |  | 5.90 (2.41) | 6.0 (2.41) | -.934 | .351 |  |
| valid N |  | | | |  | | |  |  |  |  |  | |  |  |  | 1267 | 848 |  |  |  |
| AAN = atypical anorexia nervosa; AN-R = AN restrictive subtype; AN-BP = AN binge/purge subtype; BED = binge eating disorder; BMI = body mass index; BN = bulimia nervosa; CPRS-S-A = Comprehensive Psychopathological Rating Scale – self rated version of the affective subscales; ED = eating disorder; EDE-Q = Eating Disorder Examination Questionnaire; MINI-Kid = Mini International Neuropsychiatric Interview for Children and Adolescents; OSFED = other specific feeding and EDs; SA = occurrence any lifetime suicide attempt(-s); SA recent = occurrence of any SA within the last year (only asked to those with lifetime SA); SCID = Structured Clinical Interview for DSM-IV; SDQ = Strengths and Difficulties Questionnaire; SI = occurrence of any suicidal ideation within the last three months. | | | | | | | | | | | | | | | | | | | | | |

| **Table S2.** Comparison between patients with and without complete 1-year follow-up (FU; those without FU that technically could have such data, see manuscript) assessment in relevant variables at initial registration. N for specific measures differs slightly so valid N is reported for each measure. CPRS-S-A is only available for adults; SDQ only for adolescents. Depression is based on SCID for adults and MINI-Kid for adolescents. Categorical variables examined by χ^2^, continuous measures by independent samples t-test. Effect sizes were considered small Φ>.10/ Cohen’s *d*>.20, medium Φ>.30/ *d*>.50, and large Φ>.50/ *d*>.80. Comparisons done in the whole sample (No FU N=1521; FU N=406), then separately for adults (No FU N=1023; FU N=237) and adolescents (No FU N=498; DERS N=169). Significant differences at level *p*<.05 and ≥ small effect sizes are highlighted. | | | | | | | | | | | | | | | | | | | |
| --- | --- | --- | --- | --- | --- | --- | --- | --- | --- | --- | --- | --- | --- | --- | --- | --- | --- | --- | --- |
|  | All ages | | | | |  |  |  | Adults | | |  |  |  | Adolescents | |  |  |  |
| *N, %* | No FU | | | | FU | χ^2^ | *p* | Φ | No FU | | FU | χ^2^ | *p* | Φ | No FU | FU | χ^2^ | *p* | Φ |
| SA | 258 (17%) | | | | 58 (14.4%) | 1.631 | .202 | -.029 | 210 (20.6%) | | 41 (17.3%) | 1.302 | .254 | -.032 | 48 (9.7%) | 17 (10.2%) | .039 | .844 | .008 |
| valid N | 1517 | | | | 404 |  |  |  | 1020 | | 237 |  |  |  | 497 | 167 |  |  |  |
| SA recent | 78 (30.2%) | | | | 14 (24.6%) | .726 | .394 | -.048 | 43 (20.5%) | | 7 (17.5%) | .186 | .666 | -.027 | 35 (72.9%) | 7 (41.2%) | 5.532 | **.019** | **-.292** |
| valid N | 258 | | | | 57 |  |  |  | 210 | | 40 |  |  |  | 48 | 17 |  |  |  |
| SI | 569 (37.6%) | | | | 149 (36.7%) | .101 | .751 | -.007 | 363 (35.6%) | | 77 (32.5%) | .812 | .368 | -.025 | 206 (41.6%) | 72 (42.6%) | .050 | .822 | .009 |
| valid N | 1515 | | | | 406 |  |  |  | 1020 | | 237 |  |  |  | 495 | 169 |  |  |  |
| Depression | 474 (37%) | | | | 140 (39.3%) | .658 | .417 | .020 | 289 (33.2%) | | 72 (34%) | .047 | .829 | .005 | 185 (45%) | 68 (47.2%) | .210 | .647 | .019 |
| valid N | 1282 | | | | 356 |  |  |  | 876 | | 212 |  |  |  | 411 | 144 |  |  |  |
| Males | 67 (4.4%) | | | | 15 (3.7%) | .397 | .529 | .014 | 45 (4.4%) | | 13 (5.5%) | .517 | .472 | -.020 | 22 (4.4%) | 2 (1.2%) | 3.805 | .051 | .076 |
| valid N | 1521 | | | | 406 |  |  |  | 1023 | | 237 |  |  |  | 498 | 169 |  |  |  |
| Age category distribution | | |  | | | 11.175 | **<.001** | -.076 |  | |  |  |  |  |  |  |  |  |  |
| Adults | 1023 (67.3%) | | | | 237 (58.4%) |  |  |  |  | |  |  |  |  |  |  |  |  |  |
| Adolescents | 498 (32.6%) | | | | 169 (41.6%) |  |  |  |  | |  |  |  |  |  |  |  |  |  |
| Diagnostic distribution | | |  | | | 14.009 | **.016** | .085 |  | |  | 5.448 | .364 | .066 |  |  | 18.573 | **.002** | **.167** |
| AN-R | 290 (19.1%) | | | | 105 (25.9%) |  |  |  | 143 (14%) | | 30 (12.7%) |  |  |  | 147 (29.5%) | 75 (44.4%) |  |  |  |
| AN-BP | 88 (5.8%) | | | | 22 (5.4%) |  |  |  | 60 (5.9%) | | 11 (4.6%) |  |  |  | 28 (5.6%) | 11 (6.5%) |  |  |  |
| BN | 467 (30.7%) | | | | 110 (27.1%) |  |  |  | 406 (39.7%) | | 95 (40.1%) |  |  |  | 61 (12.2%) | 15 (8.9%) |  |  |  |
| BED | 48 (3.2%) | | | | 18 (4.4% |  |  |  | 43 (4.2%) | | 18 (7.6%) |  |  |  | 5 (1%) | 0 (0%) |  |  |  |
| AAN | 295 (19.3%) | | | | 82 (20.2%) |  |  |  | 157 (15.3%) | | 36 (15.2%) |  |  |  | 138 (27.7%) | 46 (27.2%) |  |  |  |
| OSFED | 333 (21.8%) | | | | 69 (17%) |  |  |  | 214 (20.9%) | | 47 (19.8%) |  |  |  | 119 (23.9%) | 22 (13%) |  |  |  |
|  | All ages | | | | |  |  |  | Adults | | |  |  |  | Adolescents | |  |  |  |
| *Mean, SD* | No FU | | | FU | | T | *p* | *d* | No FU | | FU | T | *p* | *d* | No FU | FU | T | *p* | *d* |
| Age | 22.61 (8.37) | | | 21.97 (8.34) | | 1.359 | .174 |  | 26.20 (8.00) | | 27.04 (8.40) | -1.445 | .149 |  | 15.23 (1.28) | 14.85 (1.33) | 3.336 | **<.001** | **.291** |
| valid N | 1521 | | | 406 | |  |  |  | 1023 | | 237 |  |  |  | 498 | 169 |  |  |  |
| ED-duration | 7.39 (8.12) | | | 6.47 (7.99) | | 2.048 | .041 | **.114** | 10.17 (8.55) | | 10.08 (8.75) | .153 | .878 |  | 1.68 (1.64) | 1.40 (1.46) | 1.963 | **.050** | .181 |
| valid N | 1521 | | | 406 | |  |  |  | 1023 | | 237 |  |  |  | 498 | 169 |  |  |  |
| BMI | 21.61 (5.85) | | | 20.74 (5.72) | | 2.655 | .008 | **.149** | 22.77 (6.38) | | 22.90 (6.41) | -.294 | .769 |  | 19.23 (3.53) | 17.72 (2.32) | 6.332 | **<.001** | **.506** |
| valid N | 1521 | | | 406 | |  |  |  | 1023 | | 237 |  |  |  | 498 | 169 |  |  |  |
| Non-Accept. | 15.93 (6.26) | | | 16.24 (6.42) | | -.879 | .379 |  | 16.26 (6.20) | | 16.68 (6.64) | -.946 | .344 |  | 15.25 (6.34) | 15.61 (6.07) | -.637 | .524 |  |
| valid N | 1521 | | | 406 | |  |  |  | 1023 | | 237 |  |  |  | 498 | 169 |  |  |  |
| Goals | 16.54 (5.46) | | | 16.93 (5.15) | | -1.314 | .189 |  | 16.15 (5.38) | | 16.50 (5.21) | -.900 | .368 |  | 17.33 (5.54) | 17.54 (5.02) | -.442 | .659 |  |
| valid N | 1521 | | | 406 | |  |  |  | 1023 | | 237 |  |  |  | 498 | 169 |  |  |  |
| Impulse | 14.79 (6.42) | | | 14.74 (6.11) | | .131 | .896 |  | 14.32 (6.21) | | 14.16 (5.87) | .366 | .715 |  | 15.74 (6.74) | 15.56 (6.36) | .318 | .659 |  |
| valid N | 1526 | | | 406 | |  |  |  | 1023 | | 237 |  |  |  | 498 | 169 |  |  |  |
| Awareness | 18.37 (5.21) | | | 19.13 (5.05) | | -2.627 | **.009** | .148 | 18.19 (5.17) | | 18.64 (4.93) | -1.210 | .227 |  | 18.73 (5.26) | 19.82 (5.15) | -2.319 | **.021** | **.209** |
| valid N | 1521 | | | 406 | |  |  |  | 1023 | | 237 |  |  |  | 498 | 169 |  |  |  |
| Strategies | 21.50 (7.92) | | | 22.17 (7.84) | | -1.528 | .127 |  | 21.01 (7.69) | | 21.17 (7.61) | -.294 | .769 |  | 22.51 (8.28) | 23.58 (7.96) | -1.469 | .142 |  |
| valid N | 1521 | | | 406 | |  |  |  | 1023 | | 237 |  |  |  | 498 | 169 |  |  |  |
| Clarity | 14.05 (4.69) | | | 14.76 (4.50) | | -2.721 | **.007** | .155 | 14.03 (4.65) | | 14.13 (4.26) | -.289 | .773 |  | 14.09 (4.77) | 15.64 (4.68) | -3.667 | **<.001** | **.328** |
| valid N | 1521 | | | 406 | |  |  |  | 1023 | | 237 |  |  |  | 498 | 169 |  |  |  |
| Total Score | 101.17 (27.31) | | | 103.97 (25.55) | | -1.860 | .063 |  | 99.96 (26.70) | | 101.28 (24.98) | -.694 | .488 |  | 103.67 (28.40) | 107.75 (25.94) | -1.650 | .099 |  |
| valid N | 1521 | | | 406 | |  |  |  | 1023 | | 237 |  |  |  | 498 | 169 |  |  |  |
| EDE-Q Glob. | 3.75 (1.33) | | | 3.85 (1.23) | | -1.301 | .194 |  | 3.91 (1.17) | | 3.98 (1.03) | -.800 | .424 |  | 3.43 (1.58) | 3.66 (1.44) | -1.665 | .096 |  |
| valid N | 1521 | | | 406 | |  |  |  | 1023 | | 237 |  |  |  | 498 | 169 |  |  |  |
| CPRS-S-A depression | |  | |  | |  |  |  | 11.11 (4.71) | | 10.94 (4.19) | .486 | .627 |  |  |  |  |  |  |
| valid N |  | | |  | |  |  |  | 1023 | | 237 |  |  |  |  |  |  |  |  |
| CPRS-S-A anxiety | |  | |  | |  |  |  | 10.21 (4.39) | | 9.99 (4.23) | .661 | .508 |  |  |  |  |  |  |
| valid N |  | | |  | |  |  |  | 1023 | | 237 |  |  |  |  |  |  |  |  |
| SDQ emotional symptoms | | |  | | |  |  |  |  |  | |  |  |  | 5.96 (2.45) | 6.17 (2.30) | -.995 | .320 |  |
| valid N |  | |  | | |  |  |  |  |  | |  |  |  | 498 | 169 |  |  |  |
| AAN = atypical anorexia nervosa; AN-R = AN restrictive subtype; AN-BP = AN binge/purge subtype; BED = binge eating disorder; BMI = body mass index; BN = bulimia nervosa; CPRS-S-A = Comprehensive Psychopathological Rating Scale – self rated version of the affective subscales; ED = eating disorder; EDE-Q = Eating Disorder Examination Questionnaire; MINI-Kid = Mini International Neuropsychiatric Interview for Children and Adolescents; OSFED = other specific feeding and EDs; SA = occurrence any lifetime suicide attempt(-s); SA recent = occurrence of any SA within the last year (only asked to those with lifetime SA); SCID = Structured Clinical Interview for DSM-IV; SDQ = Strengths and Difficulties Questionnaire; SI = occurrence of any suicidal ideation within the last three months. | | | | | | | | | | | | | | | | | | | |

| **Table S3.** Suicidality at initial registration: results of logistic regression analysis. Bonferroni corrected significance level: *p*<.0125. | | | | | | | | | | | | | | |
| --- | --- | --- | --- | --- | --- | --- | --- | --- | --- | --- | --- | --- | --- | --- |
|  |  | Unadjusted | | | | | |  | Adjusted | | | | | |
| Outcome | Predictor | B | SE | Wald | *p* | OR | 95% CI |  | B | SE | Wald | *p* | OR | 95% CI |
| **Model 1** | | | | | | | | | | | | | | |
| SA | DERS tot | 0.016 | 0.002 | 56.239 | **<.001** | 1.016 | 1.012-1.020 |  | 0.011 | 0.003 | 17.763 | **<.001** | 1.011 | 1.006-1.016 |
|  | Age |  |  |  |  |  |  |  | -0.004 | 0.015 | 0.078 | .780 | 0.996 | 0.967-1.025 |
|  | ED duration |  |  |  |  |  |  |  | 0.060 | 0.015 | 16.613 | **<.001** | 1.062 | 1.032-1.093 |
|  | EDE-Q tot |  |  |  |  |  |  |  | 0.272 | 0.062 | 19.226 | **<.001** | 1.313 | 1.163-1.482 |
|  | Depression |  |  |  |  |  |  |  | -0.063 | 0.130 | 0.234 | .628 | 0.939 | 0.727-1.212 |
| SI | DERS tot | 0.027 | 0.002 | 231.023 | **<.001** | 1.028 | 1.024-1.032 |  | 0.019 | 0.002 | 76.458 | **<.001** | 1.019 | 1.015-1.024 |
|  | Age |  |  |  |  |  |  |  | -0.030 | 0.012 | 6.165 | **.013** | 0.970 | 0.947-0.994 |
|  | ED duration |  |  |  |  |  |  |  | 0.031 | 0.012 | 6.385 | **.012** | 1.032 | 1.007-1.057 |
|  | EDE-Q tot |  |  |  |  |  |  |  | 0.138 | 0.045 | 9.366 | **.002** | 1.147 | 1.051-1.253 |
|  | Depression |  |  |  |  |  |  |  | 0.626 | 0.102 | 37.642 | **<.001** | 1.870 | 1.531-2.283 |
| **Model 2** | | | | | | | | | | | | | | |
| SA | DERS non-acceptance | 0.010 | 0.011 | 0.811 | .368 | 1.010 | 0.989-1.032 |  | -0.010 | 0.012 | 0.700 | .403 | 0.990 | 0.966-1-014 |
|  | DERS goals | 0.013 | 0.016 | 0.696 | .404 | 1.013 | 0.982-1.045 |  | 0.008 | 0.018 | 0.197 | .657 | 1.008 | 0.973-1.044 |
|  | DERS impulse | 0.008 | 0.013 | 0.435 | .510 | 1.008 | 0.984-1.034 |  | 0.015 | 0.014 | 1.104 | .293 | 1.015 | 0.987-1.044 |
|  | DERS awareness | 0.027 | 0.013 | 4.419 | .036 | 1.028 | 1.002-1.054 |  | 0.022 | 0.014 | 2.279 | .131 | 1.022 | 0.994-1.052 |
|  | DERS strategies | 0.041 | 0.012 | 11.624 | **.001** | 1.041 | 1.017-1.066 |  | 0.042 | 0.014 | 9.399 | **.002** | 1.043 | 1.015-1.071 |
|  | DERS clarity | -0.20 | 0.016 | 1.602 | .206 | 0.980 | 0.949-1.011 |  | -0.031 | 0.018 | 2.899 | .089 | 0.969 | 0.935-1.005 |
|  | Age |  |  |  |  |  |  |  | -0.004 | 0.015 | 0.061 | .805 | 0.996 | 0.968-1.026 |
|  | ED duration |  |  |  |  |  |  |  | 0.061 | 0.015 | 16.932 | **<.001** | 1.063 | 1.032-1.094 |
|  | EDE-Q tot |  |  |  |  |  |  |  | 0.270 | 0.062 | 18.788 | **<.001** | 1.310 | 1.159-1.480 |
|  | Depression |  |  |  |  |  |  |  | -0.100 | 0.132 | 0.575 | .448 | 0.905 | 0.699-1.172 |
| SI | DERS non-acceptance | -0.006 | 0.009 | 0.480 | .488 | 0.994 | 0.977-1.011 |  | -0.009 | 0.010 | 0.735 | .391 | 0.991 | 0.972-1.011 |
|  | DERS goals | -0.004 | 0.013 | 0.125 | .724 | 0.996 | 0.971-1.020 |  | -0.016 | 0.014 | 1.288 | .256 | 0.984 | 0.958-1.012 |
|  | DERS impulse | 0.016 | 0.010 | 2.445 | .118 | 1.016 | 0.996-1.037 |  | 0.017 | 0.011 | 2.205 | .138 | 1.017 | 0.995-1.040 |
|  | DERS awareness | 0.054 | 0.011 | 25.977 | **<.001** | 1.055 | 1.034-1.077 |  | 0.036 | 0.012 | 9.513 | **.002** | 1.036 | 1.103-1.060 |
|  | DERS strategies | 0.091 | 0.010 | 84.961 | **<.001** | 1.096 | 1.075-1.117 |  | 0.074 | 0.011 | 45.016 | **<.001** | 1.077 | 1.054-1.100 |
|  | DERS clarity | -0.020 | 0.013 | 2.364 | .124 | 0.980 | 0.955-1.006 |  | -0.018 | 0.015 | 1.478 | .224 | 0.982 | 0.955-1.011 |
|  | Age |  |  |  |  |  |  |  | -0.027 | 0.012 | 5.046 | **.025** | 0.973 | 0.950-0.997 |
|  | ED duration |  |  |  |  |  |  |  | 0.031 | 0.012 | 6.136 | **.013** | 1.031 | 1.006-1.056 |
|  | EDE-Q tot |  |  |  |  |  |  |  | 0.124 | 0.046 | 7.371 | **.007** | 1.132 | 1.035-1.237 |
|  | Depression |  |  |  |  |  |  |  | 0.597 | 0.104 | 33.207 | **<.001** | 1.816 | 1.482-2.224 |
|  |  |  |  |  |  |  |  |  |  |  |  |  |  |  |
| Model 1-2 run on the full sample (N=2398 for SA; 2399 for SI).  Depression: current depressive episode (0=no; 1=yes); DERS: Difficulties in Emotion Regulation Scale; ED duration: eating disorder duration; EDE-Q: Eating Disorder Examination Questionnaire; SA: life-time suicide attempts (0=no; 1=yes); SI: suicidal ideation during the last three months (0=no; 1=yes). | | | | | | | | | | | | | | |

| **Table S4.** Suicidality at 1 year follow-up: results of logistic regression analysis. Bonferroni corrected significance level: p<.0125. | | | | | | | | | | | | | | | | |
| --- | --- | --- | --- | --- | --- | --- | --- | --- | --- | --- | --- | --- | --- | --- | --- | --- |
|  |  | Unadjusted | | | | | |  | | Adjusted | | | | | | |
| Outcome | Predictor | B | SE | Wald | *p* | OR | 95% CI | |  | | B | SE | Wald | *p* | OR | 95% CI |
| **Model 1** | | | | | | | | | | | | | | | | |
| SA follow-up | DERS tot | 0.032 | 0.011 | 8.679 | **.003** | 1.033 | 1.011-1.055 | |  | | -0.068 | 0.085 | 0.644 | .422 | 0.934 | 0.791-1.103 |
|  | Age |  |  |  |  |  |  | |  | | 0.020 | 0.089 | 0.050 | .823 | 1.020 | 0.857-1.214 |
|  | ED duration |  |  |  |  |  |  | |  | | 0.572 | 0.373 | 2.355 | .125 | 1.772 | 0.853-3.681 |
|  | EDE-Q tot |  |  |  |  |  |  | |  | | -1.429 | 0.763 | 3.509 | .061 | 0.240 | 0.054-1.068 |
|  | Depression |  |  |  |  |  |  | |  | | 0.001 | 0.015 | 0.004 | .950 | 1.001 | 0.972-1.031 |
|  | SA initial |  |  |  |  |  |  | |  | | 2.348 | 0.679 | 11.960 | **.001** | 10.464 | 2.766-39.592 |
| SI follow-up | DERS tot | 0.020 | 0.005 | 18.715 | **<.001** | 1.020 | 1.011-1.029 | |  | | 0.004 | 0.006 | 0.442 | .506 | 1.004 | 0.992-1.017 |
|  | Age |  |  |  |  |  |  | |  | | -0.015 | 0.030 | 0.243 | .622 | 0.985 | 0.929-1.045 |
|  | ED duration |  |  |  |  |  |  | |  | | 0.051 | 0.031 | 2.783 | .095 | 1.053 | 0.991-1.118 |
|  | EDE-Q tot |  |  |  |  |  |  | |  | | 0.138 | 0.137 | 1.017 | .313 | 1.148 | 0.878-1.503 |
|  | Depression |  |  |  |  |  |  | |  | | 0.018 | 0.293 | 0.004 | .951 | 1.018 | 0.573-1.810 |
|  | SI initial |  |  |  |  |  |  | |  | | 1.895 | 0.283 | 44.731 | **<.001** | 6.654 | 3.818-11.595 |
| **Model 2** | | | | | | | | | | | | | | | | |
| SA follow-up | DERS non-acceptance | -0.001 | 0.051 | 0.000 | .987 | 0.999 | 0.904-1.105 | |  | | -0.021 | 0.064 | .111 | .739 | 0.979 | 0.863-1.110 |
|  | DERS goals | -0.070 | 0.085 | 0.684 | .408 | 0.932 | 0.790-1.101 | |  | | -0.129 | 0.102 | 1.579 | .209 | 0.879 | 0.720-1.075 |
|  | DERS impulse | 0.120 | 0.063 | 3.615 | .057 | 1.128 | 0.996-1.277 | |  | | 0.087 | 0.077 | 1.279 | .258 | 1.091 | 0.938-1.269 |
|  | DERS awareness | 0.069 | 0.065 | 1.122 | .290 | 1.071 | 0.943-1.216 | |  | | 0.079 | 0.076 | 1.093 | .296 | 1.082 | 0.933-1.255 |
|  | DERS strategies | 0.042 | 0.052 | 0.644 | .422 | 1.043 | 0.941-1.155 | |  | | 0.025 | 0.068 | .134 | .715 | 1.025 | 0.898-1.170 |
|  | DERS clarity | 0.007 | 0.077 | 0.007 | .933 | 1.007 | 0.865-1.171 | |  | | -0.054 | 0.093 | .330 | .566 | 0.948 | 0.790-1.138 |
|  | Age |  |  |  |  |  |  | |  | | -0.055 | 0.085 | .411 | .521 | 0.947 | 0.801-1.119 |
|  | ED duration |  |  |  |  |  |  | |  | | 0.018 | 0.086 | .042 | .837 | 1.018 | 0.860-1.205 |
|  | EDE-Q tot |  |  |  |  |  |  | |  | | 0.610 | 0.381 | 2.566 | .109 | 1.841 | 0.873-3.883 |
|  | Depression |  |  |  |  |  |  | |  | | -1.322 | 0.781 | 2.863 | .091 | 0.267 | 0.058-1.233 |
|  | SA initial |  |  |  |  |  |  | |  | | 2.330 | 0.698 | 11.126 | **.001** | 10.277 | 2.614-40.402 |
| SI follow-up | DERS non-acceptance | 0.013 | 0.022 | 0.348 | .555 | 1.013 | 0.971-1.057 | |  | | 0.025 | 0.026 | 0.870 | .351 | 1.025 | 0.973-1.080 |
|  | DERS goals | 0.029 | 0.032 | 0.805 | .370 | 1.029 | 0.966-1.096 | |  | | 0.010 | 0.038 | 0.068 | .795 | 1.010 | 0.937-1.088 |
|  | DERS impulse | -0.002 | 0.026 | 0.004 | .947 | 0.998 | 0.950-1.050 | |  | | -0.049 | 0.030 | 2.580 | .108 | 0.952 | 0.897-1.011 |
|  | DERS awareness | 0.026 | 0.027 | 0.913 | .339 | 1.026 | 0.973-1.083 | |  | | -0.012 | 0.033 | 0.143 | .705 | 0.988 | 0.926-1.053 |
|  | DERS strategies | 0.058 | 0.022 | 6.777 | **.009** | 1.060 | 1.015-1.108 | |  | | 0.031 | 0.028 | 1.276 | .259 | 1.032 | 0.977-1.089 |
|  | DERS clarity | -0.034 | 0.033 | 1.058 | .304 | 0.966 | 0.905-1.032 | |  | | 0.009 | 0.040 | 0.053 | .818 | 1.009 | 0.933-1.092 |
|  | Age |  |  |  |  |  |  | |  | | -0.019 | 0.031 | 0.392 | .531 | 0.981 | 0.923-1.042 |
|  | ED duration |  |  |  |  |  |  | |  | | 0.057 | 0.032 | 3.181 | .075 | 1.058 | 0.994-1.126 |
|  | EDE-Q tot |  |  |  |  |  |  | |  | | 0.114 | 0.139 | 0.676 | .411 | 1.121 | 0.854-1.472 |
|  | Depression |  |  |  |  |  |  | |  | | 0.043 | 0.296 | 0.021 | .886 | 1.044 | 0.584-1.866 |
|  | SI initial |  |  |  |  |  |  | |  | | 1.926 | 0.955 | 42.561 | **<.001** | 6.861 | 3.847-12.238 |
| Model 1-2 run in follow-up sample (N=406). DERS: Difficulties in Emotion Regulation Scale; Depression: current depressive episode (0=no; 1=yes); ED duration: eating disorder duration; EDE-Q: Eating Disorder Examination Questionnaire; SA follow-up: any suicide attempts last 12 months (0=no; 1=yes); SA initial: life-time suicide attempts reported at initial registration (0=no; 1=yes); SI follow-up: any suicidal ideation the last three months (0=no; 1=yes); SI initial: suicidal ideation during the last three months reported at initial registration (0=no; 1=yes). | | | | | | | | | | | | | | | | |
